# Enhanced thylakoid photoprotection can increase yield and canopy radiation use efficiency in rice

**DOI:** 10.1038/s42003-018-0026-6

**Published:** 2018-03-22

**Authors:** Stella Hubbart, Ian R. A. Smillie, Matthew Heatley, Ranjan Swarup, Chuan Ching Foo, Liang Zhao, Erik H. Murchie

**Affiliations:** Division of Plant and Crop Sciences, School of Biosciences, University of Nottingham, Sutton Bonington, LE12 5RD UK

## Abstract

High sunlight can raise plant growth rates but can potentially cause cellular damage. The likelihood of deleterious effects is lowered by a sophisticated set of photoprotective mechanisms, one of the most important being the controlled dissipation of energy from chlorophyll within photosystem II (PSII) measured as non-photochemical quenching (NPQ). Although ubiquitous, the role of NPQ in plant productivity remains uncertain because it momentarily reduces the quantum efficiency of photosynthesis. Here we used plants overexpressing the gene encoding a central regulator of NPQ, the protein PsbS, within a major crop species (rice)  to assess the effect of photoprotection at the whole canopy scale. We accounted for canopy light interception, to our knowledge for the first time in this context. We show that in comparison to wild-type plants, *psbS* overexpressors increased canopy radiation use efficiency and grain yield in fluctuating light, demonstrating that photoprotective mechanisms should be altered to improve rice crop productivity.

## Abstract (web summary)

Stella Hubbart et al. show that over-expression of the photoprotective protein gene *psbS* leads to enhanced canopy-level radiation use efficiency and grain yield in rice. The results demonstrate that alterations to photoprotection and light-harvesting processes could be used to improve yield in rice crops.

## Introduction

Photosynthetic efficiency is a limitation to achieving gains in crop yield that will meet the needs of global food security in the coming century^1, 2^. However, we still lack a complete understanding of canopy photosynthesis in natural and agricultural environments. For example, plants are commonly exposed to light levels that fluctuate in time and space yet most of our understanding of photosynthesis arises from studies in static conditions.

A plethora of mechanisms regulate the amount of energy received by plant leaves and pigment protein complexes. These include plant and chloroplast movement, pigment concentration and acclimation of pigment protein complexes^3–6^. Over much shorter timescales (seconds and minutes) plants rapidly process excess absorbed light energy at the biochemical level^7,8^. One such mechanism is the inducible dissipation, or quenching, of excitation energy (measured as non-photochemical quenching, NPQ) within photosystem (PS) II. It is able to respond to sudden increases in radiation quickly and in a regulated way with minimum energetic cost to the plant^9,10^.

Quenching measured as NPQ is engaged during periods of high radiation resulting in the prompt release of chlorophyll excitation energy as heat within PSII^10,11^. It is thought to help prevent the onset of photoinhibition^12,13^. NPQ responsiveness over short timescales results from its sensitive regulation via acidification of the thylakoid lumen and the increased ΔpH between lumen and stroma^14,15^. The rate of formation and the capacity for NPQ is under control of the protein PsbS and the xanthophyll cycle^14,16–18^. PsbS was initially thought to be required for formation of the major component of NPQ, termed high-energy state quenching or qE^12,19^ but it has since been shown that NPQ can form in plants where PsbS is absent^16,20^. It seems likely that PsbS is an important regulator and accelerator of qE formation in the thylakoid membrane^9,11,20^. The xanthophyll cycle co-determines kinetics and capacity for qE and consists of the reversible formation (de-epoxidation) of zeaxanthin from violaxanthin in high light^21,22^.

qE dynamic properties are appropriate for rapid changes in the light environment. PsbS-dependent qE can be generated within seconds but the synthesis of zeaxanthin and its reconversion back to violaxanthin in low-light occurs on a timescale of minutes^22–24^ leading to the suggestion that zeaxanthin persistence is a ‘memory’ of high-light events, enabling a rapid response to a re-occurrence of saturating light^2^. Slower-relaxing components of NPQ include qI or inhibitory quenching which can be formed as a result of damage to PSII and its repair which is a process requiring time, energy and protein synthesis^25,26^ or from the more persistent retention of zeaxanthin^27^.

All components of NPQ must result in a reduction in quantum efficiency of PSII electron transport^8^ although this is unlikely to be a limiting factor during high-light periods. In particular, the impact of qI or photoinhibition on photosynthesis at whole-plant and ecological scales has been modelled and measured but empirical quantification of its effect on productivity lacks certainty^13,28,29^. One of the problems is how to measure and predict the effects caused by changing light conditions. In naturally fluctuating light qE will provide protection at high light but also limit photosynthesis in low light via the lowered quantum yield. In an attempt to quantify this, Zhu et al.^30^ used a modelling approach to show that canopy photosynthesis could be substantially reduced by the slow recovery of qI and ϕCO_2_ in low light. Similarly, Krah and Logan^31^ and Kromdjik et al.^24^ showed that qE may limit photosynthesis in fluctuating light in *Arabidopsis thaliana* and tobacco. In rice, enhanced qE directly resulted in lower photosynthesis during induction^32^. Chlorophyll fluorescence measurements in rice canopies provide evidence that NPQ results in reduced quantum efficiency at leaf level^33,34^.

Plant canopies are complex three-dimensional objects in which the light can fluctuate over short timescales by solar movement or wind, resulting in highly complex patterns^35,36^. The operation of photoprotection, therefore, sets up a cost—benefit dilemma^37,38^. qE reduces the likelihood of photoinhibition and photooxidative stress^12^ but operation in a plant canopy may reduce photosynthesis in fluctuating light. We can hypothesise that the characteristics of qE should be suited to fluctuating not static light. However most work on qE has been carried out on plants grown in static conditions^39^. Work with *Arabidopsis thaliana* plants lacking PsbS has shown that qE is important for fitness of plants in the field, however, it is unclear whether this is directly attributable to a reduction in PSII electron transport^40,41^. A severely reduced qE may have other signalling and metabolic effects on the plant^42,43^. The dynamics of both induction and relaxation of qE seem to be important^24,30^. A recent study using tobacco showed that upregulating PsbS, violaxanthin de-epoxidase and zeaxanthin epoxidase together could enhance NPQ recovery and quantum yield of CO_2_ assimilation and this was associated with increased plant biomass and yield^24^.

Photoprotection is, therefore, a significant target for crop improvement but it is necessary to understand the tradeoffs with CO_2_ assimilation and growth in fluctuating light environments. Improvements in photosynthesis per unit leaf area should result in increased growth and biomass. However, any increase in leaf area will itself enhance light capture and even if initially small can have a substantial effect on growth. To separate the effects of light ‘capture’ from the altered leaf biochemistry we should measure radiation use efficiency (RUE), which is the amount of biomass produced per unit intercepted radiation^44–46^. RUE shows a degree of variability in the field^46^ but it also responds in a predictable manner according to environmental conditions and is highly dependent on canopy photosynthesis. Canopies with high 3D complexity set-up this dilemma clearly where the frequency of large fluctuations (sunflecks) is high. A high NPQ may lead to impairment of CO_2_ assimilation during low-light or high-light induction periods^32^. On the other hand if qE is acting predominantly in a protective manner to reduce photoinhibitory costs then RUE may rise.

Here, we show that higher photoprotective capacity (via increased PsbS protein alone) levels results in enhanced biomass, RUE and grain yield in a major crop plant (rice) where biomass production is a major limitation to crop yield under fluctuating light. This is likely due to a reduction in the level of photoinhibition.

## Results

### *psbS* overexpression increases NPQ

Three glasshouse experiments (experiments 1–3) and one growth room experiment (experiment 4) were performed to measure biomass, canopy formation, NPQ levels and RUE in wild-type lines and lines overexpressing *psbS*. Plant transformation was carried out at Syngenta (Research Triangle Park, NC, USA) using an *Agrobacterium tumefaciens*-mediated technique as described previously^32^. Expression of the transgenes was driven by the Cestrum yellow leaf curling virus promoter, which contains the promoter region including the TATA box and enhancer factors. Overexpression was selected for yield studies because plants with lowered expression via RNAi were consistently smaller with low grain yield (see Hubbart et al^32^). Three glasshouse experiments were performed to measure essential parameters of growth, photosynthesis and photoprotection in the overexpression lines. A summary of the lines used and measurements made is shown in Supplementary Table 1. Experiments 1 and 2 measured biomass, leaf area, chlorophyll fluorescence, gas exchange, and were identical with the exception that RUE was measured in experiment 2 and monitoring fluorescence measurements were not made in experiment 1. Experiment 3 only measured grain yield, biomass and leaf area at harvest. Data from experiments 1 and 2 largely showed the same differences between wild-type lines and overexpression in all parameters. Experiments 1 and 2 used different lines from the T2 generation as follows (also see materials and methods): OE-33 and OE-26, experiment 3 used OE74, OE90 and OE99 and experiment 4 used OE-16. Seed availability influenced the use of different lines in each experiment. The overexpression lines had consistently higher NPQ throughout and we combined data from these lines to compare with wild-type lines. Gene expression analysis of these lines showed higher expression of PsbS in all lines, in comparison to the wild-type plants (Supplementary Fig. 1). A previous paper showed substantially higher amounts of PsbS in overexpression lines^32^ but this could not be measured in this study. Here, we use the gene expression levels to indicate higher PsbS protein content.

Data from experiment 2, 3 and 4 is shown in Figs. 1–8. Data from experiment 1 and showing values for individual lines is presented in Supplementary Figs. 1–9.

### Overexpression lines show higher biomass and leaf area

**Fig. 1:**
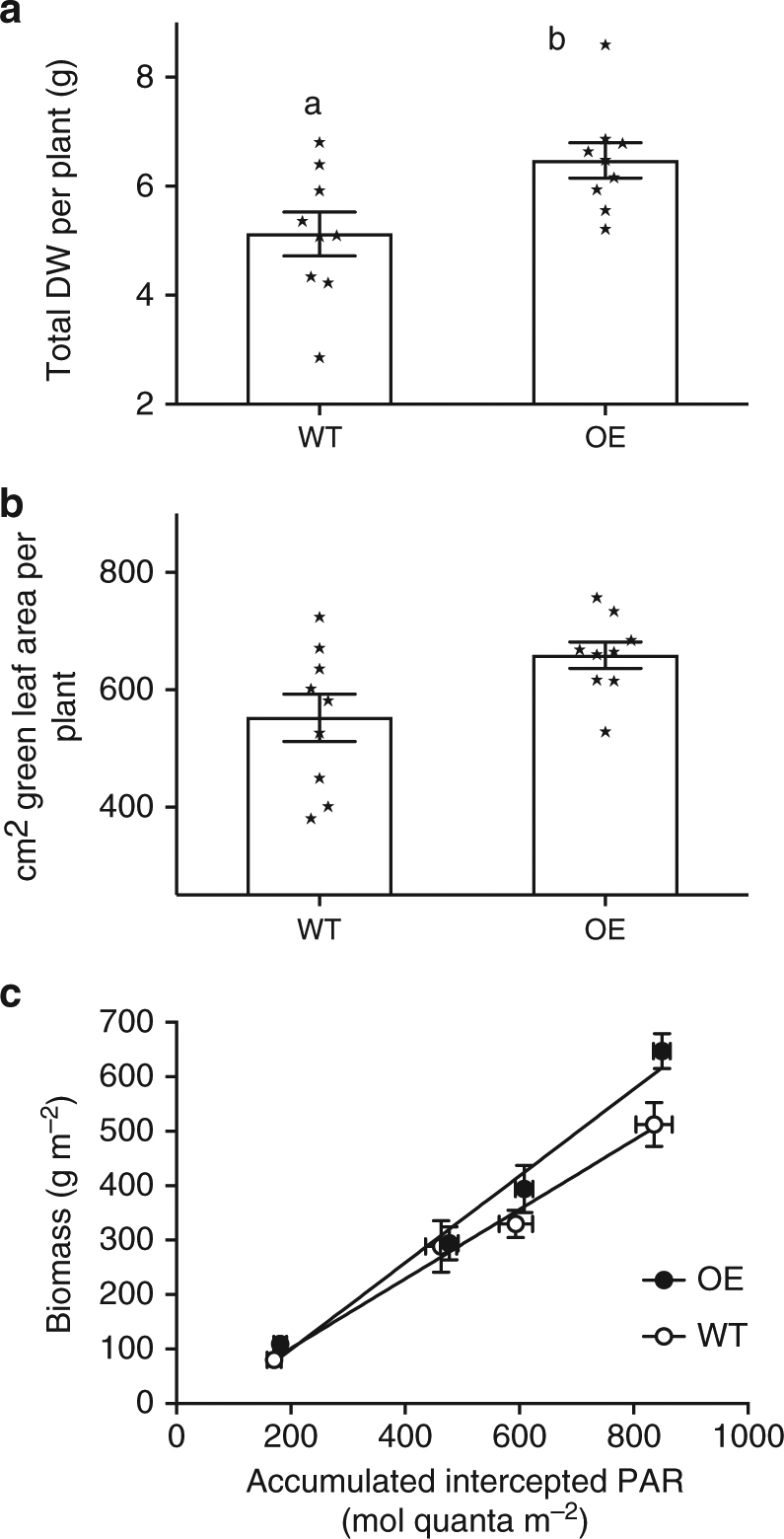
Overexpression of *psbS* is associated with higher radiation use efficiency. **a** Biomass at leaf 9 stage, **b** leaf area at leaf 9 stage and **c** Radiation use efficiency (RUE) for the 4 weeks preceding leaf 9 stage calculated by plotting canopy accumulated intercepted radiation and accumulated above ground dry weight (biomass). Data from experiment 2 is shown. Regression lines are shown and used to calculate RUE, which is given by the slope between the two variables. Values are average ± standard errors of the means of each plot i.e., for **a**, **b** *n* = 9 (means of measurements from individual plants from all plots) and for **c** *n* = 3 (wild-type, WT) and *n* = 6 (overexpressors, OE) (means of plot means). Letters ‘a’ and ‘b’ indicate significant difference (*p* = 0.019, unpaired *t*-test)

The impact of overexpression of important photoprotective regulator genes such as *psbS* on biomass and RUE in major crop plants is not known and here we investigate using canopy analysis techniques. Figure 1 and Supplementary Fig. 2 shows dry weight (DW) and leaf area (measured destructively) of the canopy at leaf 9 stage. There was a significantly higher (*p* = 0.019, unpaired *t*-test) value for DW (6.5 g per plant) in the overexpressors compared to the wild-type lines (5.1 g per plant) and significantly higher final leaf area (Supplementary Fig. 2). To confirm the higher biomass for overexpression lines during the canopy formation stage in experiment 2 we measured RUE over a 4-week period leading up to the formation of leaf 9 (Fig. 1c). This process normalises biomass production against intercepted radiation. Again we see the higher biomass for overexpression lines at leaf 9 stage but the slope of this relationship gives the RUE, which was 22% higher in overexpression lines than wild-type lines (0.8 compared to 0.64 kg DW mol quanta intercepted^1^). Therefore, the conversion of intercepted radiation to biomass was higher in the overexpression lines for the period of measurement.

### Overexpression lines have higher grain yield

**Fig. 2:**
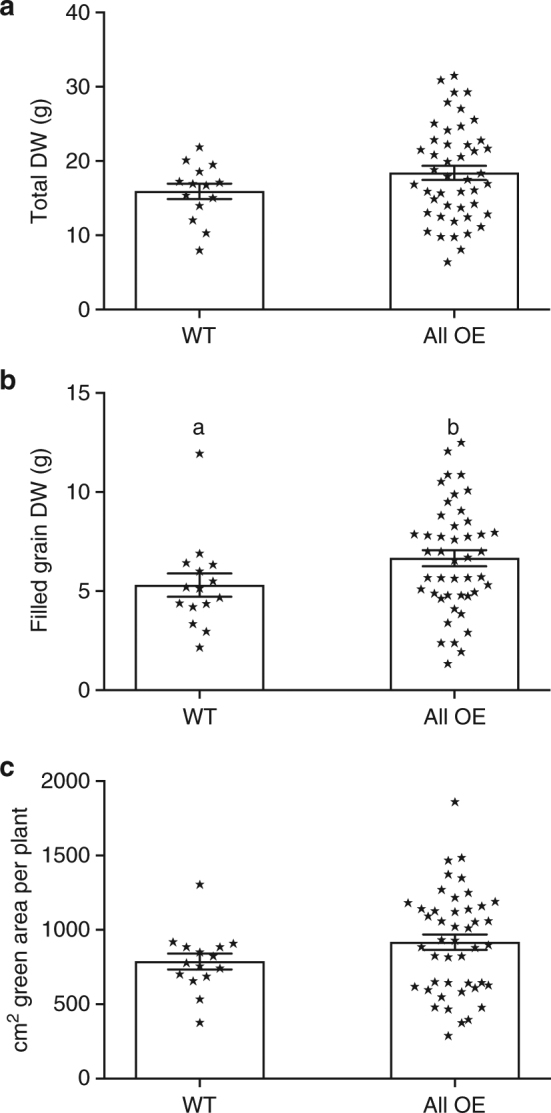
Overexpression of *psbS* is associated with higher grain yield and biomass at harvest. Grain yield and biomass at harvest in wild-type (WT) and overexpression (OE) lines. **a** Total above ground plant dry weight (DW), **b** filled grain DW per plant, **c** green area per plant. Values are means ± standard errors of the means for a single experiment (means of measurements from individual plants from all plots) (*n* = 14 (WT) and 42 (OE). Data from experiment 3 is shown. Letters ‘a’ and ‘b’ indicate significant difference (*p* = 0.0874), unpaired *t*-test)

Higher biomass and RUE does not necessarily drive a higher yield and can depend upon efficient partitioning into grain. Figure 2 shows grain yield, green leaf area and final DW at harvest following grain ripening. Overexpressing lines had a 26% higher grain yield (*p* = 0.0874, unpaired *t*-test) compared to wild-type lines but not any other parameter.

**Fig. 3:**
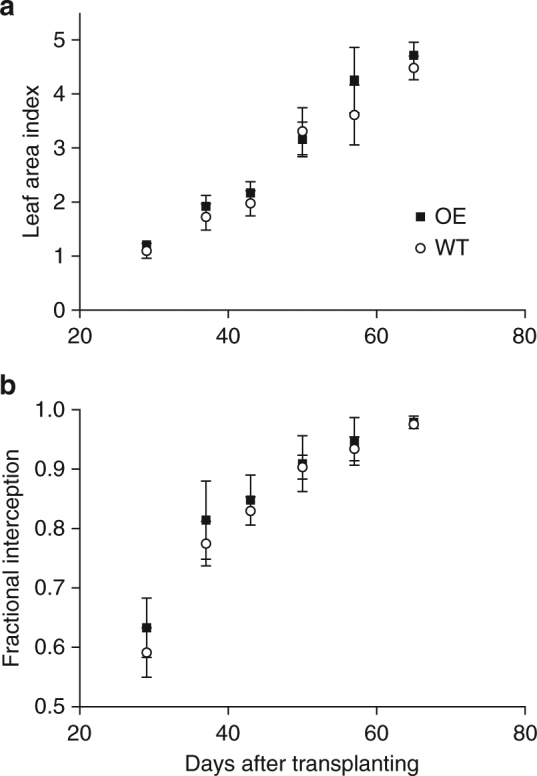
Light interception is not altered by overexpression of *psbS*. Leaf area index (LAI) and fractional interception (*F*), combining the data for the two overexpression (OE) lines in Experiment 2. Values are means of plot means ± standard errors of the means. For *F*, giving weekly values per plot. Data from experiment 2 is shown. *n* = 3 (WT) and 6 (OE). No significant differences were found (*p* < 0.05) between OE and WT for each weekly calculation

Figure 3 and Supplementary Fig. 3 shows no difference between wild-type lines and overexpression lines with respect to the rate of increase in leaf area index (LAI) nor fractional interception (*F*) by the canopy, indicating that the amount of light captured by each canopy was the same throughout development.

### NPQ dynamics increase biomass in PsbS overexpression lines

**Fig. 4:**
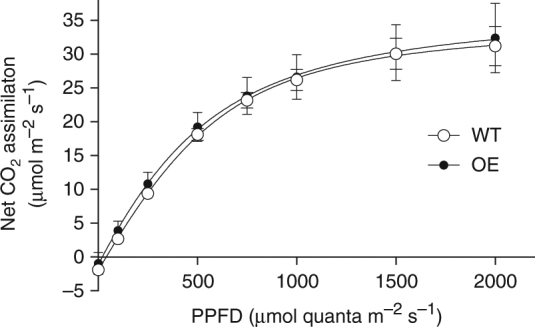
Light response curves of photosynthesis are not altered by overexpression of *psbS*. Light response curves of photosynthetic CO_2_ assimilation, combining the data for the two overexpression (OE) lines in experiment 2. Measurements were made at approximately 40 days after transplanting over a 3-day period at 30 ^o^C, a cuvette [CO_2_] of 400 ppm and ambient humidity levels. Lines shown were fitted using a non-rectangular hyperbola^6^. Values are means ± standard errors of the means (means of measurements from individual plants from all plots), *n* = 4

What is the origin of the higher biomass, RUE and grain yield in the overexpression lines? To test the photosynthetic capabilities of the plants, light response curves were measured on the newest fully expanded leaf (Fig. 4 and Supplementary Fig. 4). No differences were observed in any parameter (Amax, quantum yield, convexity or light compensation point), consistent with Hubbart et al.^32^, which used the same rice lines grown in CE chambers.

**Fig. 5:**
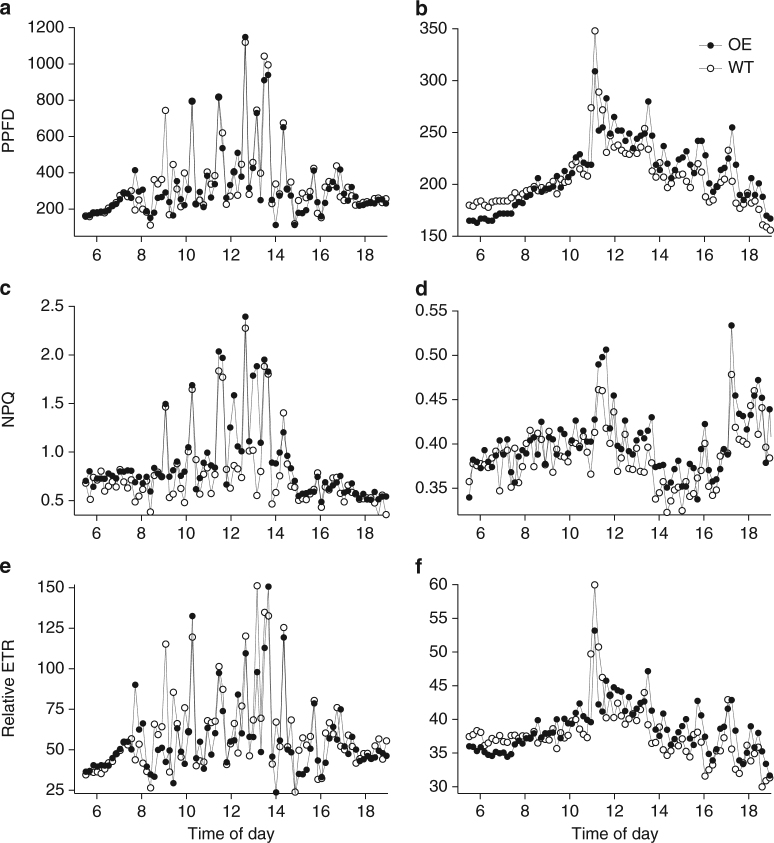
Examples of a daily course in chlorophyll fluorescence monitoring. Time course of PPFD (photosynthetic photon flux density), NPQ and relative electron transport rate (ETR) measured on a typical sunny day (**a**,** c**,** e**) and a typical cloudy day (**b**,** d**,** f**) measured using the Monitoring PAM fluorometers. Wild-type (WT) and overexpressing (OE) line OE-33 were used. Single data points are shown. Measurements were made every 10 min

**Fig. 6:**
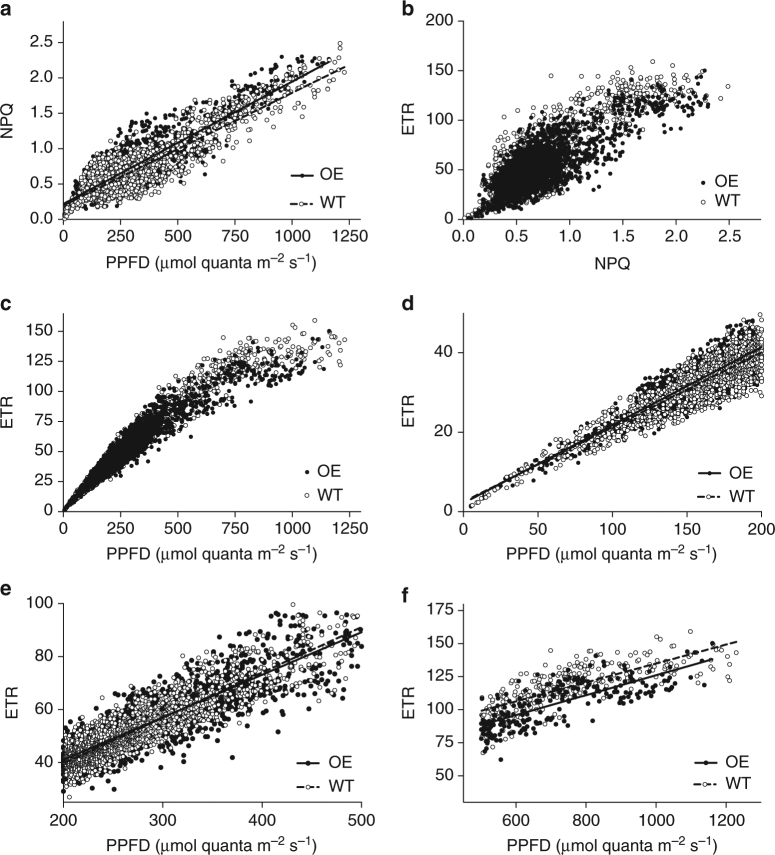
Regression analysis of chlorophyll fluorescence monitoring data show differences in electron transport rates between wild-type and *psbS* overexpressors. Comparisons between NPQ, relative electron transport rate (ETR) and PPFD (photosynthetic photon flux density) using all of the Monitoring Pam data collected over a 14-day period up to the formation of leaf 9 on the main stem in experiment 2. Single measurements shown (wild-type (WT) and the two overexpressing (OE) lines). Two sensors were used for WT measurements and one each for the two OE lines, changing plant every 3 days. Data from experiment 2. These data were used in a regression model (Table 1 and Supplementary Table 1) to show significant differences in the relationship between NPQ, ETR and PPFD for OE and WT lines. Here, we highlight key differences by separating each portion of the curve and showing linear regression lines for **a** the entire NPQ vs. PPFD response, **d** the linear light-limited portion of the ETR vs. PPFD response (0–200 µmol  m^−2^ s^−1^), **e** the intermediate portion (200–500 µmol  m^−2^ s^−1^) of the ETR vs. PPFD response and **f** the high region of the ETR vs. PPFD response ( > 500 µmol  m^−2^ s^−1^). The intermediate PPFD range **e** shows the transition between the low range (where ETR is higher in the OE) and the high range (where ETR is higher in the WT). Slopes (WT and OE, respectively) are **a** 0.001591 and 0.001729, **d** 0.1881 and 0.1963, **e** 0.1713 and 0.1608, **f** 0.07155 and 0.07526. We point out that these lines are for the purpose of statistical analysis only and have not been forced through zero. They cannot, for example, be used as a direct calculation of quantum yield

To further analyse the mechanism, Fig. 5 and Fig. 6 examine the hypothesis that the dynamics of NPQ and photosynthesis in response to light could be responsible for the enhanced biomass in overexpression lines. Figure 5 shows in detail measurements of PPFD (photosynthetic photon flux density), NPQ and relative ETR made during a typical high-light day (long periods of full sun) and a typical low-light day (long periods of cloud cover) using the monitoring chlorophyll fluorescence technique. This figure shows clearly that the light was rarely static, with frequent and fast switches between high and low light and overexpression lines often showing higher values than the wild-type lines. The magnitude of NPQ increase when comparing wild-type lines to overexpression lines was between 7% and 100%, consistent with that seen in Hubbart et al.^32^. For the days shown, NPQ in the overexpression lines was higher than wild-type lines 80% of the time (high-light day) and 75% (low-light day). (See also Supplementary Fig. 5, Table 1 and Supplementary Table 2). Figure 6 analyses this in depth and shows all the data collected by monitoring chlorophyll fluorescence, plotting PPFD against NPQ and relative ETR and NPQ against ETR. The large number of data points makes visual comparison difficult but a number of issues are clear. First the majority of data points are from PPFD below 500 μmol m^−2^ s^−1^, i.e., below light saturation. Second the NPQ–PPFD relationship is more linear than ETR–PPFD. Lastly, there was a tendency for relative ETR to be higher in the wild-type lines at the higher PPFD levels (above 500 μmol m^−2^ s^−1^).

**Table 1:** Summary of regression analysis performed on NPQ (non-photochemical quenching) and relative ETR (electron transport rate) using monitoring chlorophyll fluorescence

PPFD band	vs. PPFD	*p*-value for slope differences between WT and OE	*p*-value for displacement of parallel lines between WT and OE	Predicted value at mean PPFD	
				WT	OE
< 200 µmol m^−2^ s^−1^	Relative ETR (log_10_)	< 0.001	< 0.001	1.476 ± 0.0019	1.489 ± 0.0018
	NPQ	< 0.001	0.003	0.475 ± 0.0036	0.505 ± 0.0035
200–500 µmol m^−2^ s^−1^	Relative ETR (log_10_)	< 0.001	< 0.001	1.715 ± 0.0011	1.719 ± 0.0011
	NPQ	0.415	0.01	0.608 ± 0.0036	0.684 ± 0.0036
> 500 µmol m^−2^ s^−1^	Relative ETR (log_10_)	0.056	< 0.001	2.062 ± 0.0029	2.024 ± 0.0028
	NPQ	< 0.001	< 0.001	1.440 ± 0.0135	1.552 ± 0.0129

See Fig. 6 and Supplementary Table 1 for graphical display and full statistical output. Data was divided into three PPFD (photosynthetic photon flux density) bands: <200 µmol  m^−2^ s^−1^; 200–500 µmol  m^−2^ s^−1^; >500 µmol m^−2^ s^−1^. Regression and analysis of variance was performed using Genstat (VSN International). Following a test for homogeneity of variance, log_10_ relative ETR was used. Estimates of slope and displacement show differences between overexpression lines (OE) and wild-type (WT) with a significance threshold at < 0.05. The predicted value of relative ETR and NPQ is taken at the mean PPFD value for each PPFD band

In order to analyse the differences in photosynthesis between the overexpression lines and wild-type lines, a regression analysis was performed on the NPQ and ETR data. Data sets were divided into bands according to PPFD region to address the possibility that different mechanisms will place a limitation on photosynthesis according to the state of light (saturation or limitation). Although such analyses should be treated with a little caution when using them to predict a mechanism, the fact that a large number of measurements has been made over a long-time period in a fluctuating environment with a high sampling frequency allows important hypotheses to be tested. For example, recovery from photosynthesis could be expected to be important at low light^24^, whereas limitation during high light could be imposed by high PsbS levels^32^. The regression analysis is shown in Table 1 and Supplementary Table 1.

Following tests for homogeneity of variance, Log10 ETR was used. Here, we tested for a significant difference between the slopes of each response for overexpression lines and wild-type lines. We then tested for significant displacement between overexpression lines and wild-type lines after adjusting for the slope value. In all three PPFD bands (0–200 μmol m^−2^ s^−1^, 200–500 μmol m^−2^ s^−1^ and above 500 μmol  m^−2^ s^−1^), NPQ in overexpression lines was significantly higher than in wild-type lines (*p* < 0.001, analysis of variance). ETR values showed a different pattern, being significantly higher in wild-type lines than overexpression lines at PPFD above 500 μmol m^−2^ s^−1^ but higher in overexpression lines at the lower PPFD bands in comparison to wild-type lines. We point out that these lines are for statistical purposes only, have not been forced through zero and cannot, for example, be used as direct calculation of quantum yield. To test whether enhanced PsbS levels reduce onset of photoinhibition, dark adapted Fv/Fm was measured at mid-day on two of the sunniest days to estimate the level of photoinhibition on the newest fully expanded leaf. Taking an average across both days, values for overexpression lines and wild-type lines, respectively, (means ± standard error of means) were 0.797 ± 0.01 and 0.765 ± 0.01 (*p* = 0.0403, unpaired *t*-test) showing that photoinhibition levels were lower in the overexpression lines.

### High PsbS and NPQ levels are beneficial in fluctuating light

**Fig. 7:**
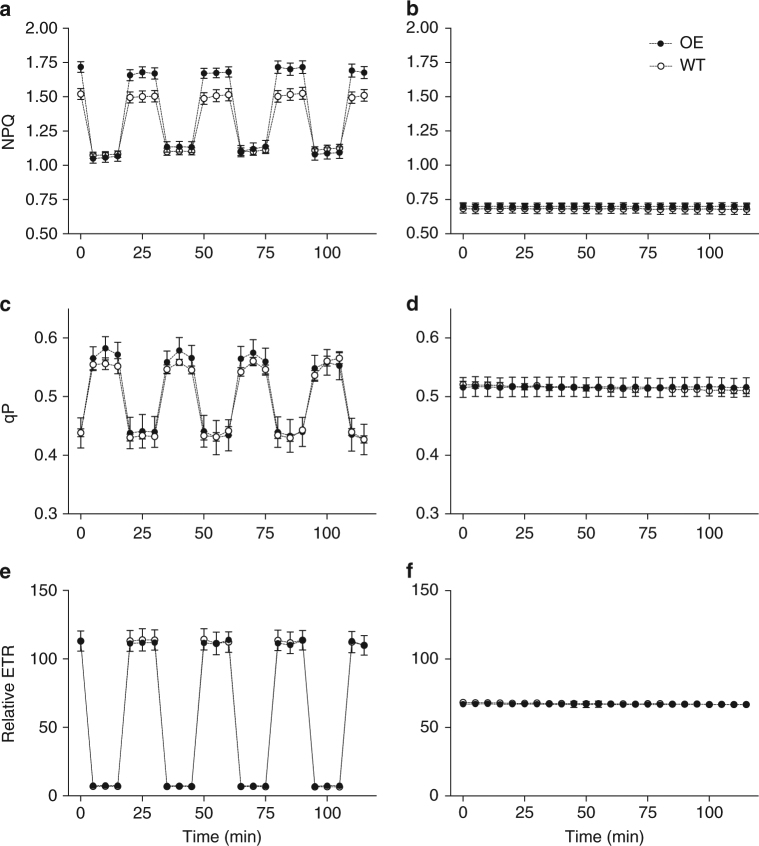
Artificially induced fluctuations in growth light reveal the photoprotective effect of *psbS* overexpression in rice. An example of NPQ, qP and relative ETR measured within artificially fluctuating light (**a**,** c**,** e**) and static light (**b**, **d**, **f**) for overexpressing (OE) and wild-type (WT) lines. Light fluctuated within the Fytoscope (PSI, Brno, Czech Republic) cabinet between 1500 and 100 µmol m^−2 ^s^−1^ every 3 min or was kept static at 800 µmol photons m^−2^ s^−1^ during the light phase of a 12 h photoperiod. The total daily PPFD was the same for both treatments. To clearly demonstrate the differences between lines and treatments a 2 h portion during mid-day is shown over a 3-day period during the experiment. Three sensors were used each for WT and OE. The Monitoring Pam pulses (applied every 5 min) and the light fluctuations were not synchronised. Values are average ± standard errors of the means from two independent experiments. *n* = 18 (3 moniPAM heads per plant type)

The characteristics of the light environment that endowed an advantage to the psbS overexpression lines are not clear. It is hypothesised that higher protective capacity may be an advantage in fluctuating light conditions so an experiment was conducted where light was the sole variable. To do this a controlled environment room was used that was capable of rapid and continual adjustment of irradiance levels. Figure 7 demonstrates the responses of NPQ, qP and ETR over a typical mid-day period. Higher NPQ in all lines and all times was induced in the fluctuating treatment compared to non-fluctuating. Overall, the magnitude of differences in relative ETR and qP between lines was substantially less than NPQ suggesting a level of regulation over PSII electron transport.

**Fig. 8:**
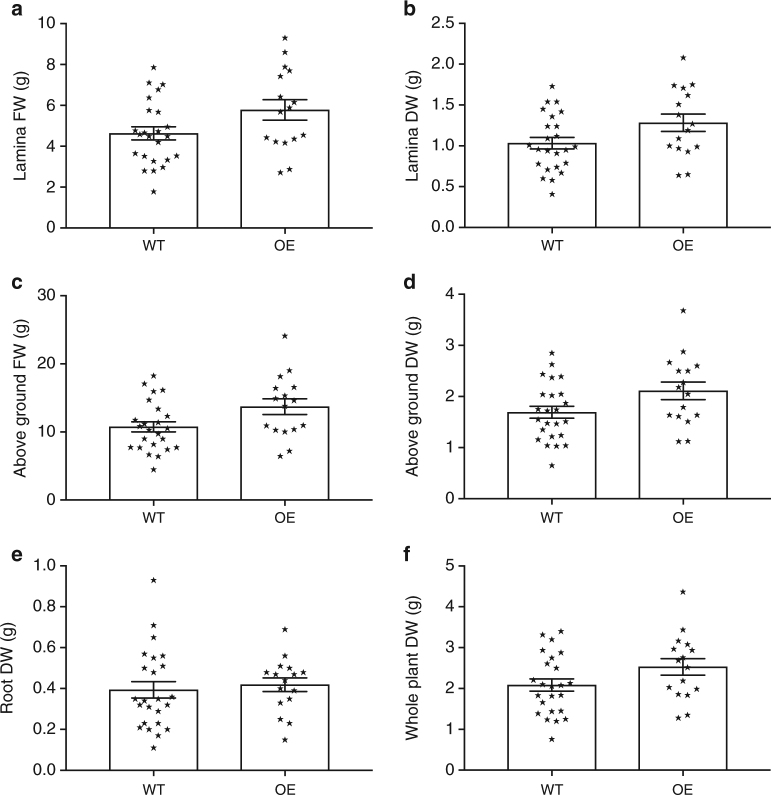
Biomass is higher in psbS overexpressors than wild-type when grown in artificially fluctuating growth light. Harvest analysis of wild-type (WT) and overexpression (OE) (3–16) lines grown under artificially fluctuating light (**a**) leaf lamina fresh weight (FW), **b** leaf Lamina dry weight (DW), **c** Above ground FW, **d** Above ground DW, **e** root DW, **f** Whole plant (root + shoot + stem (not shown) DW). Values are means of individual plants from all plots ± standard errors of the means for two independent experiments (*n* = 24 (WT) and 16 (OE)). *p*-values (unpaired *t*-tests) were as follows For lamina FW, Lamina DW, Above ground FW, Above ground DW, Root DW and Whole-Plant DW, *p* = 0.0506, 0.0476, 0.0289, 0.0423, 0.6614, 0.0804)

Figure 8 and Supplementary Fig. 10 shows harvest data from the controlled fluctuating light experiment. The important comparisons were made under the fluctuating treatment where the harvest fresh weight (FW) and above ground plant DW of the overexpressing lines was consistently and significantly higher than the wild-type lines plants in all parameters except root DW and whole-plant DW (*p* = 0.6614 and 0.0804, unpaired *t*-test). In the static light treatment where the only difference was whole-plant DW (*p* = 0.0804, unpaired *t*-test). Interestingly, wild-type plants had a slightly lower biomass and leaf area in static compared to fluctuating conditions. We conclude that high PsbS and NPQ levels are beneficial for carbon gain in the fluctuating light but not necessarily in static light.

## Discussion

Plants possess many mechanisms that can act in a photoprotective manner and be readily measured in controlled conditions. However, there are practical difficulties when extending this to quantification of productivity in natural settings and so there has been a reliance on modelling to make predictions^13,30,47–49^. To demonstrate an unequivocal role in yield improvement in the field, qE must be genetically manipulated and tested in natural conditions. It is also necessary account for the fact that changes in leaf area and canopy light interception (caused themselves by enhanced leaf photosynthesis) can incrementally exaggerate the impact of the leaf level process on biomass and yield. However, this condition has not generally been met when assessing the impact of photoprotection on plant productivity.

The use of a major crop species like rice rather than model species such as *Arabidopsis* is important because future grain yield increase in rice is known to be largely limited by biomass production and hence RUE^1,2^. The finding that enhanced photoprotection increases rice biomass and yield is largely consistent with our understanding that qE represents a substantial proportion of the energy processed by photosynthetic systems^50^. Here, we also achieved the same effect using challenging artificially fluctuating conditions, confirming that the fluctuating nature of the environment is critical and implying that enhancement of qE benefits CO_2_ assimilation. Enhanced qE was also associated with higher mid-day ${\rm Fv}/{\rm Fm}$ indicating that alleviation of photoinhibition and associated reduction in quantum yield of photosynthesis was a factor affecting biomass. It is assumed that the reduction in quantum yield that occurs as a result of photoinhibition will result in a lowered photosynthesis during low-light periods^51^ but measurements of gas exchange were not capable of exposing this possibly because the differences were too small over short time periods. We did not observe improvements of light-induced dynamics of CO_2_ assimilation^24^ possibly because the changes in CO_2_ assimilation were too small when measured individually and instrumentation insufficiently sensitive. So although no phenotype in terms of dynamics of CO_2_ assimilation or NPQ dynamics could be shown here, further conclusive work is required and should investigate other features such as xanthophyll cycle dynamics and linear vs. cyclic electron transport. Related to this point, previous work on some of the lines used in this study found no differences in relative thylakoid protein levels (sodium dodecyl sulphate polyacrylamide gel electrophoresis) [32] although further detailed analysis of thylakoid component stoichiometry is necessary.

Here, we hypothesise that small improvements in electron transport and photosynthesis over a large leaf area in the upper to middle portions of the canopy especially was sufficient to result in a substantial improvement in RUE at the whole canopy scale. Elevated qE could reduce photoinhibition by prevention of over-reduction of PSII and lowered levels of reaction centre inactivation. Alternatively qE has been implicated in reducing levels of reactive oxygen species^52,53^ and this may affect the repair rate of PSII following photodamage^25^. The differences in NPQ observed between wild-type and overexpression lines were small in comparison with previous work^32^ and this is likely due to the fact that we measured in situ in a glasshouse rather than at saturating irradiance. However, it also raises the possibility that another effect of PsbS protein was responsible for the differences in biomass and yield (see below).

PsbS also enhances the rate of recovery from qE in low light^54^ in the presence or absence of zeaxanthin albeit over much shorter timescale in comparison to zeaxanthin retention. Assuming that ϕCO_2_ recovery is also enhanced in PsbS over-expressors this provides an additional or alternative explanation for the enhanced biomass. We used chlorophyll fluorescence monitoring to integrate responses across a long-time period (Figs. 5 and 6). Interestingly, the high levels of PsbS in overexpression lines resulted in a lower ETR in high-light periods, which is consistent with the observations of Hubbart et al.^32^, who concluded that enhanced qE partially inhibits photosynthesis during the transition to high-light periods. This suggests that the majority of photosynthesis contributing to yield arose from leaves in a light-limited rather than light-saturated state, consistent with the observations of Kromdjik et al.^24^. Thus, we see key differences: first ETR was significantly higher in overexpression lines at PPFD values below 500 μmol m^−2^ s^−1^. This could arise either from enhanced recovery from qE or reduction in onset of sustained quenching resulting from photoinhibition. However, the overexpression lines showed higher NPQ under low light (in which qE and qI cannot be distinguished) and this would tend to reduce ETR. This apparent paradox may be could be resolved if the effect of overexpression of PsbS is to limit the photoinhibitory rise in Fo (the minimum fluorescence yield in a dark-adapted state). Hence ETR and NPQ can rise while ${\rm Fv}/{\rm Fm}$ falls. A photoinhibitory rise in Fo has been observed in rice in the field previously^34^.

We conducted experiments in a glasshouse using supplementary lighting in an English summer, where typical irradiance maxima were <1500 μmol m^−2^ s^−1^. This is lower than would be experienced in rice growing regions closer to the equator but still high enough to periodically saturate photosynthesis and induce photoinhibition. It is possible that the effects observed here were due to the more frequent occurrence of light-limited rates of photosynthesis. We see a similar impact on biomass and yield to Kromdjik et al.^24^ who also overexpressed PsbS but in the same plants enhanced the rate of recovery from and the rate of formation of qE in tobacco by overexpressing violaxanthin de-epoxidase and zeaxanthin epoxidase. Following the data in the current study, it will be necessary to isolate the role of each of these genes in terms of plant growth. Since PsbS enhances recovery of low-light recovery over short timescales much shorter timescales in comparison to zeaxanthin retention^24,54^ we suggest that the effect observed in the current experiment is largely due to reduction in onset of sustained quenching or the Fo rise rather than rates of recovery.

The developing canopy may place progressively different demands on the requirements for photoprotection. The effect on RUE was notable during canopy formation where the low LAI and fluorescence intensity (*F*) will result in a high proportion of the leaf surface area being exposed to higher light and the effect of ROS or photoinhibition will have a proportionally greater effect on plant photosynthesis and biomass production. As LAI and *F* rise, leaves in the lower portions of the canopy will have a lower risk of photoinhibition making an interesting comparison with dynamics of light tolerance during *Arabidopsis* development.^55^

During canopy formation light dynamics as a result of shifting self-occlusion patterns induced by solar movement would become progressively more complex resulting in an increased cost due to onset and recovery of qE and qI^24,30^ and photosynthesis induction^32,33,56^. The data in this paper suggests that high PsbS protein levels conferred an advantage in low light and a disadvantage in high light. There may also be a simple effect of self-protection of lower leaves by the upper leaves in the canopy. The overexpression lines had higher leaf area than the wild-type lines towards the end of the experiment: we cannot completely rule out the possibility that the higher leaf area resulted in biomass production not accounted for by the measurement of fractional interception.

Energy dissipation at the canopy level has not been studied in detail but it is now clear that this needs to be done if we are to improve the functioning of qE in crops. One answer would be to simply screen for high NPQ in a variety of crop germplasm such as mutants and introgression lines^57,58^. But is there genetic variation for qE in important crop species? The first quantitative genetic analysis in *A. thaliana* concluded that variation in qE could not be linked to PsbS^59^. However, the same is not true for rice: recent significant work^60^ showed that the presence of a *PsbS* allele was responsible for the difference in qE between japonica and indica genotypes. It is possible that the presence of an association in rice but not *A. thaliana* represents adaptation to high-light environments in the former and the importance of qE as a component of RUE in crops such as rice.

We hypothesise that optimisation of canopy photoprotection is influenced by both the LAI and the precise canopy architecture, which will determine the penetration of direct sunlight and light dynamics^30,35,61,62^. Open canopies or those with upright leaves or lower LAI will require a higher qE capacity, higher rate of qE induction in addition to enhanced recovery. The manipulation of photosynthetic properties according to canopy position has been suggested previously^63^ but not in relation to photoprotection. It may be appropriate to link the expression of photoprotective genes to conditions of high light or to the high-light acclimation response^64,65^. Such approaches provide a fascinating route towards the improvement of crop yield.

An improvement in crop photosynthetic efficiency at the leaf and canopy level is required to achieve a step-change in yield to meet global food security targets. Primary photosynthetic events are central to growth and development but have not yet been specifically exploited in agriculture or plant breeding. Here we show for the first time, to our knowledge, in a major crop species that enhancements to biomass and grain yield are possible by alterations to light harvesting and energy processing by increasing the capacity for qE.

## Methods

### Glasshouse experiments: growth of plants and experimental design

Experiments took place between May and October of 2012, 2013 and 2015 in a south-facing glasshouse at Sutton Bonington campus, University of Nottingham (52°49’59”N, 1°14’50”W) designed for the growth of crop stands within a controlled environment. It consisted of a concrete tank 5 x 5 x 1.25 m positioned at ground level. The tank was filled entirely with a sieved sandy loam soil extracted from local fields. Plants were provided with adjusted levels of macro and micronutrients, following soil analysis. Watering took place via automated trickle tape application to maintain field capacity throughout. Supplementary sodium lighting was supplied at a position of approximately 3 m above ground level and was regulated via a light sensor external to the glasshouse such that it was activated only when external irradiance fell below approximately 200 μmol m^−2^ s^−1^ and inactivated when this was exceeded. A time delay of approximately 15 min was used before switching back on. In this way periods of low light were supplemented and excessive combinations of sunlight and artificial lighting avoided. Photoperiod in the glasshouse was regulated to 14 h using automated black out blinds. Temperature in the glasshouse was regulated to 30 ± 3 ^o^C by automated venting and two gas-fired boilers. Humidity in the glasshouse was not regulated and varied between 60 and 70%.

Transgenic rice lines were generated by Syngenta, NC, USA using the variety Kaybonnet as described^32^. Previous work confirmed higher PsbS protein levels and NPQ in these lines^32^. The homozygous T2 generation was used in these experiments. Previous work has shown that loss or reduction in qE in natural conditions may induce pathways associated with stress and defence and result in lower fitness and biomass^41,43^. For this reason, we focus on overexpression where the low qE phenotype is not apparent. We saw no differences between wild-type lines in overexpression lines in terms of key morphological events.

Seeds were germinated in the glasshouse in module trays using Levington’s seed and modular compost (Everris plc, Ipswich UK) and transplanted to the plots at the appearance of leaf 3. A complete randomised plot design was used with a 10 cm spacing between adjacent plants and a 1 x 1 m plot size (therefore plots consisted of 10 plants x 10 plants square) with 10 cm spacing between adjacent plots and a single access path 30 cm wide through the centre of the plots. A double row of wild-type plants was planted around the entire experiment to prevent any edge effects. Each plant type (wild-type and overexpression lines) had three replicate plots, randomly positioned.

Three glasshouse experiments took place: in experiment 1 a single destructive harvest for biomass and leaf area was made at the leaf 9 stage. Non-destructive measurements of photosynthesis, NPQ, canopy fractional interception and LAI were made during growth (see below). In experiment 2, rice plants were grown as in experiment 1 but a protocol was added for the measurement of canopy RUE as described below. Experiment 3 only measured biomass, leaf area and grain yield components following ripening, which was not possible in 1 and 2. All experiments used the same replicated, randomised plot arrangement. Supplementary Table 1 summarises the lines used in each experiment and the measurements that were made.

### Canopy and photosynthesis analysis

In experiments 1 and 2, radiation at the top of the plots was measured using four PPFD (photosynthetic photon flux density) line quantum sensors (Skye, Llandrindod Wells) evenly spaced across the top of the experiment approx. Ten centimetre above plant height. Data was logged every 10 min using the glasshouse software control system. In experiments 1 and 2, measurements of canopy fractional interception (*F*) and LAI were made weekly (experiment 2) or twice weekly (experiment 1) by placing a hand-held line ceptometer (Accupar LP80, Pullman WA) across the base of the canopy at ground level (*I*) and at the top of the canopy (Io). An average of 3–4 readings was taken to obtain the value of *I* and Io for each plot. Fractional interception was calculated as $({\rm Io} - I)/{\rm Io}$. Accumulated intercepted radiation was calculated using data from the line quantum sensor PPFD levels for the days preceding the measurement.

RUE in experiment 2 was measured by extracting 2 plants on a weekly basis from each plot and immediately measuring DW as described^32^. To minimise disruption to other measurements an extracted plant was never next to an adjacent extracted plant and extraction was restricted to one half of each plot and only made on five occasions. From comparisons with control plots where no extraction was made we found that extraction did not affect the measurements of fractional interception.

In experiment 3, plants were harvested above ground and split into leaf, stem and panicle fractions. Five plants were extracted from each plot. Green area (leaves + stems) was determined for individual leaf and stem fractions using a Licor area metre (Li3100c, Licor, Nebraska) while grain number was determined for individual panicles per plant. The fresh and DWs were then determined for each plant component; before DW leaves and stems were first dried at 80 ^o^C for 48 h while panicles and grains were first dried at 40^o^C for 24 h (to maintain viability and reduce water content to 10–13%).

At the start of experiments 1 and 2, individual plants from each plot were screened for NPQ with a Fluorcam (Photon Systems Instruments, Brno, Czech Republic) using the protocol described in Hubbart et al.^32^. Ten plants with highest NPQ in each plot were tagged for photosynthesis measurements in each plot. Typically we found that over 90% of leaves in overexpression line plots showed NPQ values that were higher than the wild-type lines average value. To measure photosynthesis continually over a substantial period of growth Monitoring Pam fluorometer sensors (Moni-head 485, Walz, Effeltrich, Germany) were deployed for a period of 2 weeks during canopy expansion. These were connected to two data collection devices (MoniDA, Walz, Effeltrich, Germany). These were clipped to the most recently fully expanded mature leaf on the main tiller and positioned such that the leaf light sensor was oriented at approximately 45^o^ vertically and facing in a southerly direction. This position most accurately represented the natural angle of the leaves, according to leaf curvature it was the surface most commonly exposed to the sun. A saturating pulse of blue light was applied every 10 min and values of PPFD, *F* and Fm*’* (maximum fluorescence yield in a light-adapted state) recorded to calculate non-photochemical quenching (NPQ), operational yield of PSII (ϕPSII), photochemical quenching (qP) and electron transport rate (ETR). NPQ was calculated using equation $({\mathrm {Fm}}-{\mathrm {Fm}}')/{\mathrm {Fm}}'$, where the previous night-time value of Fm (maximum fluorescence yield in a dark-adapted state) was utilised. The highest night-time value of Fm was used in each case. A pulse interval of 10 min minimised the effects of progressive Fm quenching during the night. To avoid undue stress on the leaf caused by the leaf clip and to ensure that the canopy was sampled extensively, the leaf was changed to another previously tagged plant in the same plot every three days. Fo’ was calculated using Fm*’* and the night-time measurement of Fm using the method of Oxborough and Baker (1997). Relative ETR was calculated from ϕPSII (assuming a leaf absorbance of 0.84 and PSII/PSI of 0.5)^66^.

In experiment 1, NPQ was also measured using a Fluorcam (Photon Systems Instruments, Brno, Czech Republic) at a single point early in canopy development exactly as described in Hubbart et al^32^.

Photoinhibition was measured as dark adapted Fv/Fm at mid-day using a Walz (Effeltrich Germany) MiniPam fluorometer and a 30-min dark adaptation period^32^. This measurement was made on all plots for two days where radiation was relatively high (see Fig. 5).

Photosynthesis light response curves were taken using a Licor6400XT (LI-COR, Nebraska) when the plants reached leaf seven stage. Measurements were made on non-dark-adapted plants between the hours of 9.00 a.m. and 3.00 p.m. on a cloudy day. Block temperature was 30 °C, cuvette [CO_2_] was 400 ppm, humidity was ambient (scrubber was off) and flow rate was 500 ml min^−1^. Light was provided by a combination of in-built red and blue LEDs. Illumination occurred over a series of six photosynthetically active radiation values between 2000 and 50 μmol m^−2^ s^−1^, moving from high to low, with a minimum of 2 min and maximum of 3 min at each light level.

### Artificially fluctuating light

Rice plants were grown in a FytoScope FS-SI 3400 chamber (Photon Systems Instruments, Brno, Czech Republic) under red, blue and FR light-emitting diodes (LED) diodes. It was operated on a 12/12-h photoperiod, 28 °C ambient temperature and a 55% relative humidity. Irradiance was provided by a panel of LEDs inlaid into ceiling as a sole light source with a programmed fixed blue/red light ratio of 100:75 and a constant day time background of FR ( < 5 µmol  m^−2^ s^−1^). PPFD was measured routinely at the level of the top of canopy. Plants were grown with the same procedure and hydroponics system described previously^32^. PsbS transgenic lines (OE 3-16) had six replicates and wild-type control had eight replicates planted in six hydroponic containers (considered as six blocks) in uniform distribution. The positions of hydroponic containers were changed every 2 days. The experiment was performed twice.

For germination, the growth chamber was set to 500 µmol  m^−2^ s^−1^ PPFD,12/12-h photoperiod, 28 °C ambient temperature and a 55% relative humidity. Seven days later (three weeks after germination at leaf 3 stage), the plants were treated using a fluctuating light programme keeping other parameters the same. The day time programme was: from 0 to 2 h, light ramped linearly from 0 to 500 µmol m^−2^ s^−1^; 2 to 10 h, lights fluctuated between 1500 and 100 µmol  m^−2^ s^−1^ with 3 min at each intensity; from 10 to 12 h, lights switched to 500 µmol  m^−2^ s^−1^ and then immediately ramped linearly down to 0. The lights were off for 12 h until the next day time loop. In the control programme, the settings were identical except that a constant 800 µmol m^−2^ s^−1^ were maintained from 2 to 10 h. Total daily applied PPFD was identical for both treatments. Moni-PAMs (Walz, Effeltrich, Germany) were used as described above for a 3-week period with the exception that pulses were made every 5 min due to the rapid fluctuations. Following three weeks of growth within this environment, entire plants were harvested. Leaf area and DW of individual plants were measured as described above.

Experiments in this paper were analysed as randomised block (growth room) or complete randomised design (glasshouse experiments 1–3). Prism v 7.0 (Graphpad Software Inc.) was used for curve fitting and to apply unpaired *t*-tests. Genstat (VSN International) was used for regression and analysis of variance.

### Data availability

The source data from this paper is available at https://figshare.com/s/b7e266150f8471d9c007. The material from this study is available on reasonable request and subject to a satisfactory material transfer agreement with Syngenta, Inc.

## Electronic supplementary material

Supplementary Information(PDF 515 kb)
