# Supplementary material for: Enhanced thylakoid photoprotection can increase yield and canopy radiation use efficiency in rice
Source: Commun Biol. 2018 Mar 22;1:22. doi: 10.1038/s42003-018-0026-6 (PMC6123638; doi:10.1038/s42003-018-0026-6)
Supplement: Supplementary file 1 — Supplementary Information(PDF 515 kb) [file 42003_2018_26_MOESM1_ESM.pdf]

Supplementary Table 1. A summary of the three experiments carried out including the lines used and the measurements made.

Experiment no.	Location	Lines used	Types of measurement made
1	Glasshouse	OE3-26,OE3-33	Biomass at leaf 9 stage, Leaf area at leaf 9 stage, photosynthesis light response curves, chlorophyll fluorescence: non photochemical quenching (NPQ) screen using a Fluorcam (Photon systems instruments, Brno, Czech Republic).
2	Glasshouse	OE3-26,OE3-33	Biomass, Leaf area, radiation use efficiency (biomass produced per unit radiation intercepted) up to leaf 9 stage, photosynthesis light response curves, chlorophyll fluorescence: NPQ using a monitoring fluorometer (Walz, Effeltrich, Germany)
3	Glasshouse	OE3-74,OE3-90,OE3-99	Total plant biomass, filled grain yield per plant, green leaf area, Stem area, NPQ using a monitoring fluorometer (Walz, Effeltrich, Germany)
4	LED Growth room (fluctuating light vs non-fluctuating light)	OE3-16. RNAi4-02	Chlorophyll fluorescence (monitoring fluorometer Walz, Effeltrich, Germany), biomass, leaf area

Supplementary Table 2. Regression analysis for the MoniPAM monitoring data.

Data was divided into PPFD regions 200 μmol m^{-2} s^{-1} PPFD, 200 – 500 200 μ mol m^{-2} s^{-1} PPFD, > 500 μ mol m^{-2} s^{-1} PPFD (PPFD = photosynthetically active variation). Following a test for homogeneity of variance, log_{10} relative ETR was used.

	**Estimates of PPFD parameters**					**Accumulated analysis of variance**					
**Log10ETR vs PPFD** <200 μmol m^{-2} s^{-1} PPFD	Response variate:	logetr (LT200 PPFD)									
	Fitted terms:	Constant + PPFD + planttype + PPFD.planttype									
	Parameter	estimate	s.e.	t(2673)	t pr.	Change	d.f.	s.s.	m.s.	v.r.	F pr.
	Constant	0.94369	0.00568	166.06	<.001	+ PPFD	1	47.802431	47.802431	10225.33	<.001
	PPFD	0.0035554	0.0000351	101.19	<.001	+ planttype	1	0.122358	0.122358	26.17	<.001
	planttype WT	-0.01353	0.00264	-5.12	<.001	Residual	2673	12.496021	0.004675		
						Total	2675	60.420810	0.022587		
**NPQ vs PPFD** <200 μmol m^{-2} s^{-1} PPFD	Response variate:	NPQ (LT200 PPFD)									
	Fitted terms:	Constant + PPFD + planttype + PPFD.planttype									
	Parameter	estimate	s.e.	t(2672)	t pr.	Change	d.f.	s.s.	m.s.	v.r.	F pr.
	Constant	0.2142	0.0147	14.57	<0.001	+ PPFD	1	9.58347	9.58347	564.40	<.001
	PPFD	0.0018910	0.0000933	20.26	<0.001	+ planttype	1	0.59447	0.59447	35.01	<.001
	planttype WT	**0.0633**	0.0212	2.99	0.003	+ PPFD.planttype	1	0.34809	0.34809	20.50	<.001
	PPFD..planttype WT	**-0.000607**	0.000134	-4.53	<0.001	Residual	2672	45.37004	0.01698		
						Total	2675	55.89607	0.02090		
**Log10ETR vs PPFD** 200-500 μmol m^{-2} s^{-1} PPFD	Response variate:	logetr (200 to 500 PPFD)									
	Fitted terms:	Constant + NPQ + planttype + NPQ.planttype									
	Parameter	estimate	s.e.	t(3660)	t pr.	Change	d.f.	s.s.	m.s.	v.r.	F pr.
	Constant	1.38528	0.00474	292.31	<.001	+ PPFD	1	24.879635	24.879635	11316.67	<.001
	PPFD	0.0011990	0.0000162	74.09	<.001	+ planttype	1	0.016746	0.016746	7.62	0.006
	planttype WT	**-0.03799**	0.00681	-5.58	<.001	+ PPFD.planttype	1	0.056810	0.056810	25.84	<.001
	PPFD.planttype WT	**0.0001211**	0.0000238	5.08	<.001	Residual	3660	8.046490	0.002198		
						Total	3663	32.999682	0.009009		
**NPQ vs PPFD** 200-500 μmol m^{-2} s^{-1} PPFD	Response variate:	NPQ (200to500)									
	Fitted terms:	Constant + PPFD + planttype + PPFD.planttype									
	Parameter	estimate	s.e.	t(3660)	t pr.	Change	d.f.	s.s.	m.s.	v.r.	F pr.
	Constant	0.3081	0.0156	19.70	<.001	+ PPFD	1	29.64743	29.64743	1238.13	<.001
	PPFD	0.0013481	0.0000534	25.24	<.001	+ planttype	1	5.16744	5.16744	215.80	<.001
	planttype WT	**-0.0576**	0.0225	-2.57	0.01	+ PPFD.planttype	1	0.01588	0.01588	0.66	0.415
	PPFD.planttype WT	**0.0000641**	0.0000786	-0.81	0.415	Residual	3660	87.64007	0.02395		

						Total	3660	122.47083	0.03343		
**Log10ETR vs PPFD** >500 μmol m^{-2} s^{-1} PPFD	Response variate: logetr (>500 PPFD)<br>Fitted terms: Constant + PPFD + planttype + PPFD.planttype										
	Parameter	estimate	s.e.	t(496)	t pr.	Change	d.f.	s.s.	m.s.	v.r.	F pr.
	Constant	1.7885	0.0131	136.46	<.001	+ PPFD	1	1.425677	1.425677	706.61	<.001
	PPFD	0.0003157	0.0000176	17.96	<.001	+ planttype	1	0.182737	0.182737	90.57	<.001
	planttype WT	**0.0713**	0.0176	4.05	<.001	+ PPFD.planttype	1	0.007379	0.007379	3.66	0.056
	PPFD.planttype WT	**-0.0000442**	0.0000231	-1.91	0.056	Residual	496	1.000740	0.002018		
						Total	499	2.616533	0.005244		
**NPQ vs PPFD** >500 μmol m^{-2} s^{-1} PPFD	Response variate: NPQ (>500 PPFD)<br>Fitted terms: Constant + PPFD + planttype + PPFD.planttype										
	Parameter	estimate	s.e.	t(496)	t pr.	Change	d.f.	s.s.	m.s.	v.r.	F pr.
	Constant	-0.0787	0.0605	-1.30	0.194	+ PPFD	1	53.78249	53.78249	1250.51	<.001
	PPFD	0.0021843	0.0000811	26.92	<.001	+ planttype	1	1.49685	1.49685	34.80	<.001
	planttype WT	**0.2750**	0.0814	3.38	<.001	+ PPFD.planttype	1	1.01744	1.01744	23.66	<.001
	PPFD.planttype WT	**-0.000519**	0.000107	-4.86	<.001	Residual	496	21.33220	0.04301		
						Total	499	77.62898	0.15557		

Supplementary figure 1. Gene expression levels of *psbS*.

a)

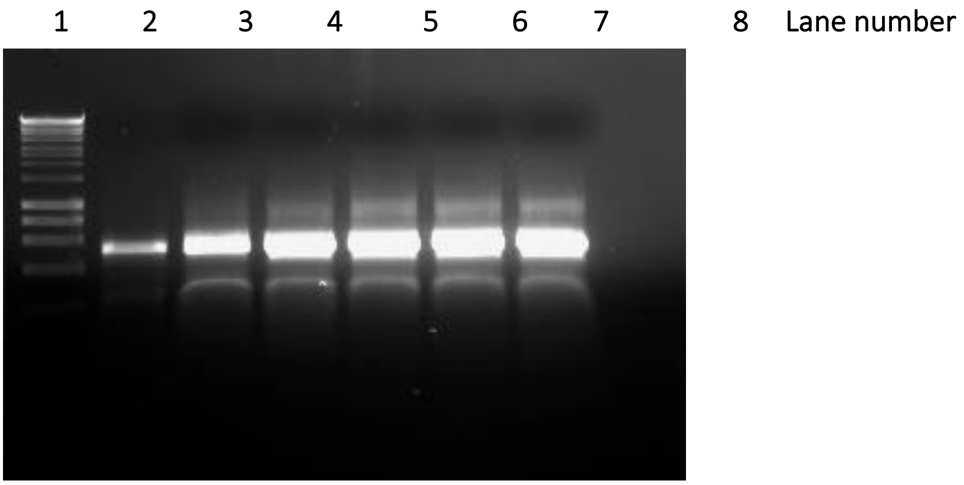

b)

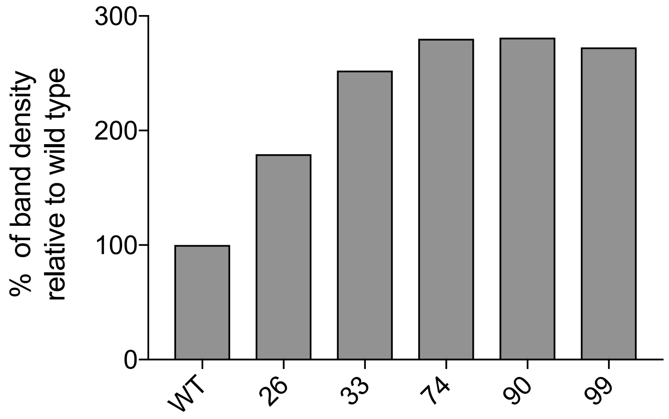

a) Expression levels of *psbS* in all lines. Lanes , left to right above: 1 – Hyperladder 1KB (Bioline, London, UK); 2 – WT Rice reference: Kaybonnet; 3 – Rice OE26; 4 – Rice OE33; 5 – Rice OE74; 6 – Rice OE90; 7 – Rice OE99; 8 – Negative control (water). RNA extraction was carried out using traditional Trizol-chloroform method. Plants were grown hydroponically in a controlled environment room as in Hubbart et al (2012)[1] at 600 mmol m^{-2} s^{-1} PPFD, 30 °C, 12 hr photoperiod and approx. 50 % humidity. Leaf samples from leaf 5 were collected in 1.5mL Eppendorf tubes at mid – day and ground to fine powder in liquid nitrogen. One leaf was collected per plant and the gel shows the result fro m a single leaf. 1000µL trizol and 200µL added to each sample. The mixture was incubated on ice for ten minutes, with frequent vigorous mixing. The samples were centrifuged at 13000 rpm at 4°C for 15 minutes. Clear supernatant was collected and an equal amount of isopropanol was added. The mixture was mixed well by inversion and incubated at -20°C overnight. The samples were centrifuged again at 13000 rpm at 4°C for 15 minutes. Pellet was retained and washed with 70% ethanol before drying and resuspended in 30µL RNAse free water. DNAse treatment was carried out using Precision DNase Kit (Primer Design,

Southampton, UK). RNA is stored at -80°C. cDNA synthesis was carried out using Superscript III Reverse Transcriptase (Invitrogen, California, US).

RT-PCR was set up using the synthesized cDNA as template. RT-PCR was carried out in a thermocycler GeneAmp® PCR System 9700 (Applied Biosystems; California, USA) for PCR amplification under the following optimized conditions: initial denaturation of one cycle at 95 °C for 4 minutes, 40 cycles of denaturation at 95 °C for 30 seconds (strand separation), 54 °C for 30 seconds (annealing) and 72 °C for 1 minute 30 seconds (extension) and one cycle of final extension at 72 °C for ten minutes. Finally, the reaction was held at 15 °C.

The PCR products were resolved using gel electrophoresis. Using RP6 and RP7 primers, expected band size is 530bp. RP6: 5'-CGG CTC GAT CTC GTT GAT-3'. RP7: 5'-CAA GAA AGG CTG AGC CGA AG-3'

b) Quantitative analysis of signal intensity of the *psbS* band using Image J software v1.50i (National Institute of Health, USA). The density of each band was quantified using the gel analyser functions within the software and expressed as a % of the value of the wild type

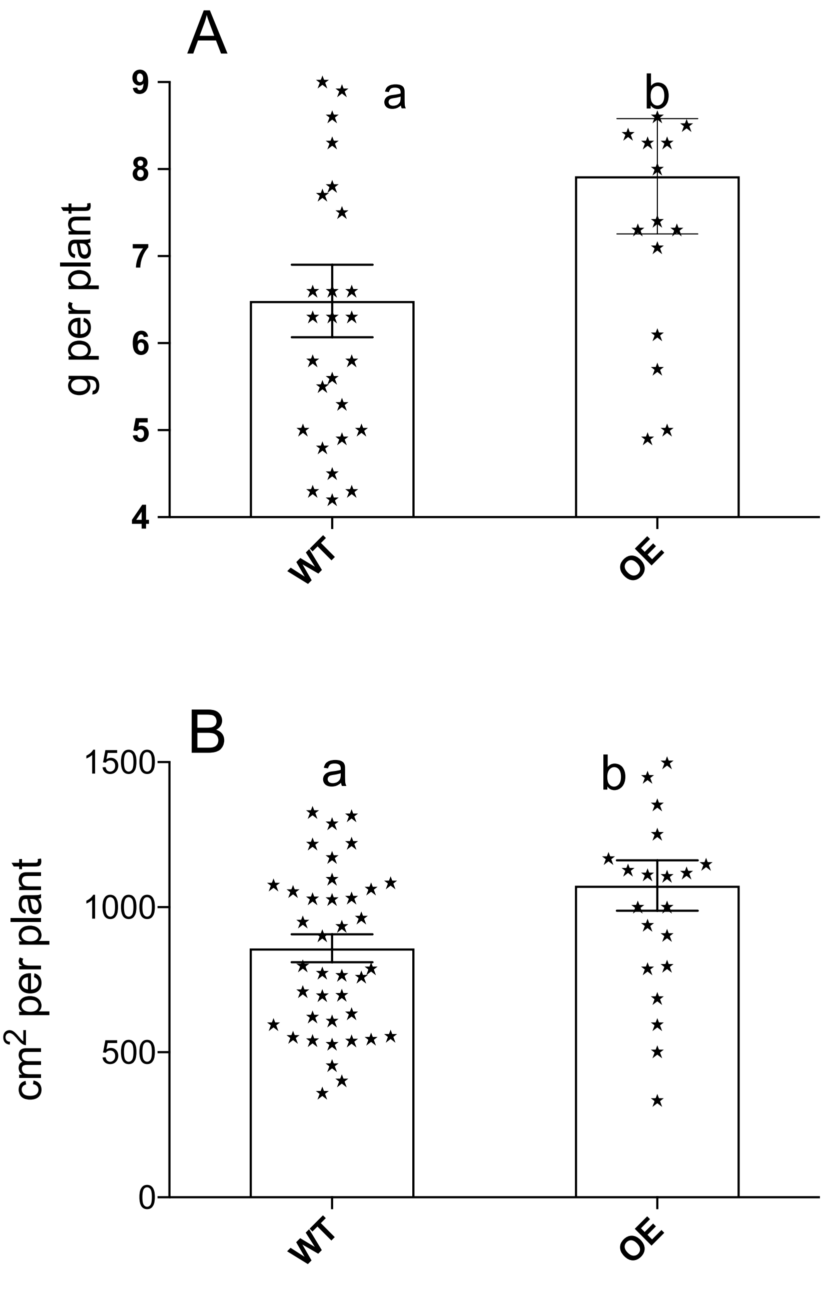

Supplementary Figure 2. Biomass and Leaf area in experiment 1 in *psbS* over expressors and wild type rice. (A) Biomass at leaf 9, (B) leaf area at leaf 9. Means of individual plants for all plots and standard error of the means are shown, n=40 (wild type, WT) and 30 (over expressing, OE) for experiment 1. Data for the two OE lines were combined Letters 'a' and 'b' indicate significant difference (p=0.0598 and 0.0208 for biomass and leaf area respectively, unpaired T tests).

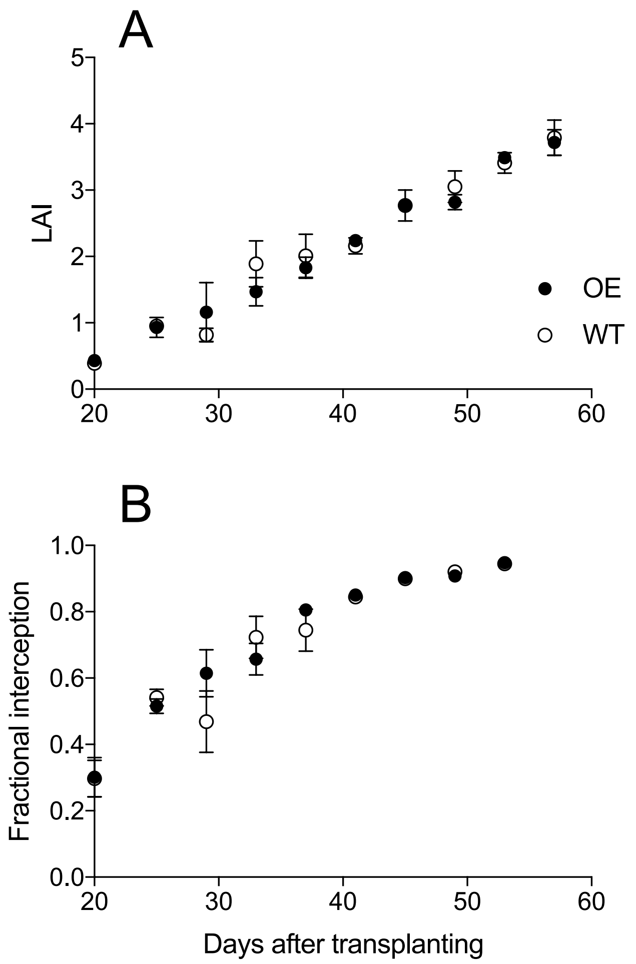

Supplementary Figure 3. Leaf area index and fractional interception in experiment 1 in *psbS* over expressors and wild type rice. Leaf area index (LAI) and fractional interception (F) for experiment 1. Data for the two overexpressing (OE) lines were combined. Values are plot means ± standard errors of the means. For F, giving twice weekly values per plot, n=3 (WT) and n=6 (OE). No significant differences were found between OE and wild type (WT) for each weekly calculation (P<0.05).

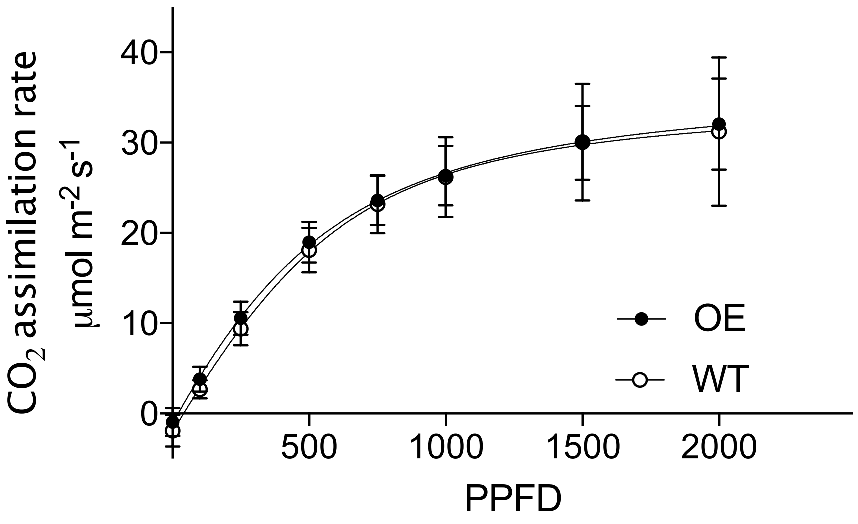

Supplementary Figure 4. Light response curves of photosynthetic CO_2 assimilation for experiment 1. Data for the two overexpressing (OE) lines were combined. Measurements were made at approximately 40 days after transplanting over a three-day period at 30 °C, a cuvette [CO_2] of 400 ppm and ambient humidity levels. Lines shown were fitted using a non-rectangular hyperbola [6]. Values are means of individual plants from all plots ± standard errors of the means (n=4).

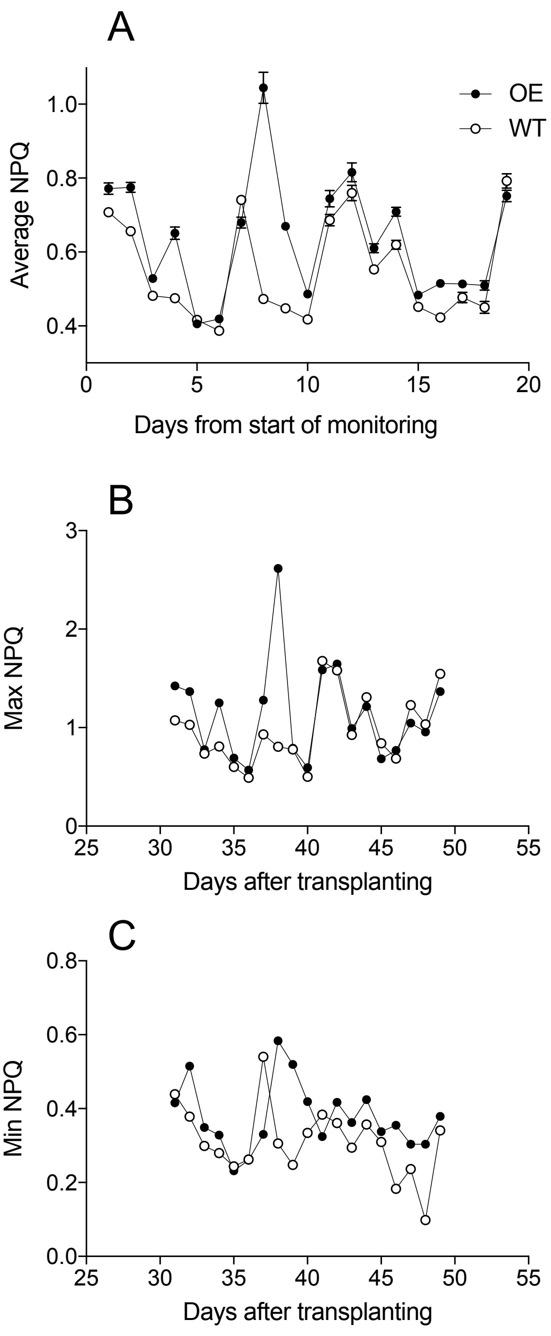

Supplementary Figure 5. Photoprotection measured over a three week period in *psbS* over expressors and wild type rice. Non-photochemical quenching measured at leaf level (MoniPam, Walz, Effeltrich, Germany) for a three week period during canopy formation in experiment 2. Measurements were made every 10 minutes over a continuous 24 hour period. Values were derived from an average of all measurements taken during the light following the removal of the blackout blinds (13 hour photoperiod). We combine the data for the two overexpressing (OE) lines. Calculation of NPQ as described in materials and methods. Values are means ± standard errors of the means.

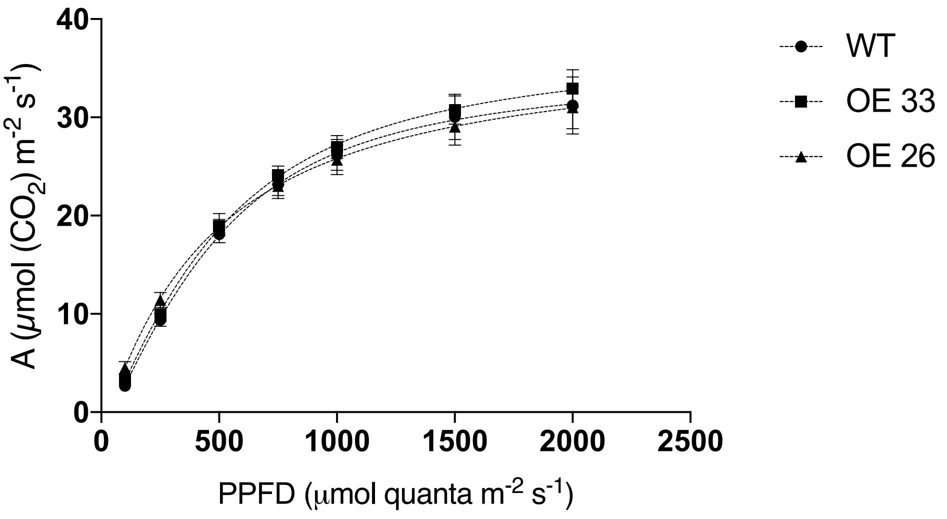

Supplementary Figure 6. Light response curves of photosynthesis in individual lines in experiment 1.. Data for individual lines from experiment 1 showing light response curves, leaf area index and fractional interception. Values shown are plot means and standard errors of the means (n=3) and there were no significant differences between wild type (WT) and over expressing (OE) lines (P<0.05).

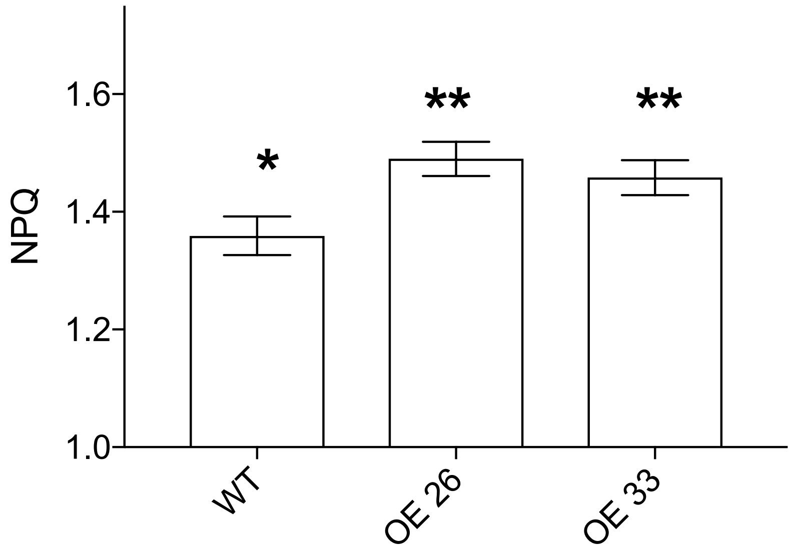

Supplementary Figure 7. Photoprotection in individual lines for experiment 1 in *psbS* over expressors and wild type rice. Data for individual lines from experiment 1, showing non photochemical quenching measured using a Fluorcam (Brno Photon Systems instruments, Czech Republic) as described in Hubbart et al (2012[37]). Overexpressing (OE) and wild type (WT) values shown are means from individual plants across all plot and standard errors of the means, n=3. Asterisks indicate a significant difference (P = 0.0068 and 0.0477 when comparing wild type and OE26 and OE33 respectively, one way analysis of variance).

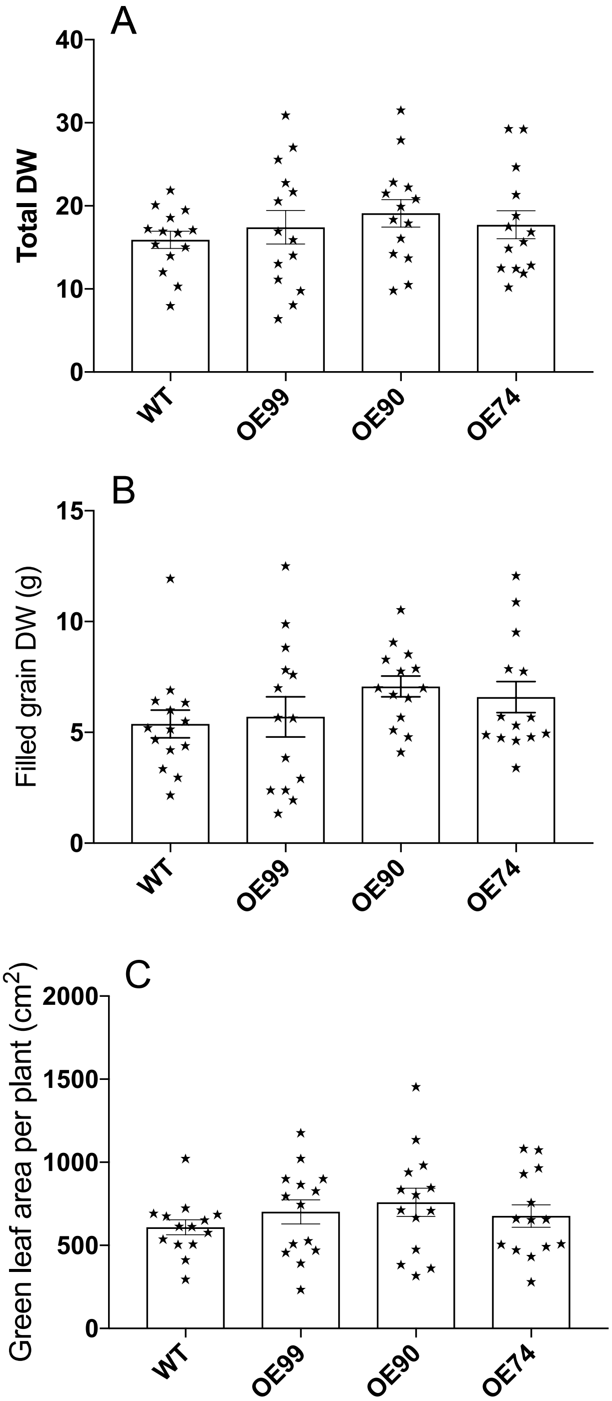

Supplementary Figure 8. Yield and biomass for individual lines in experiment 3 in *psbS* over expressors and wild type rice. Data for individual lines from experiment 3 where (A) total dry weight, (B) filled grain yield and (C) green leaf area per plant was measured. Overexpressing (OE) and wild type (WT) values shown are means of measurements from individual plants from all plots, n=14 and there were no significant differences between wild type and over expressing lines(P<0.05).

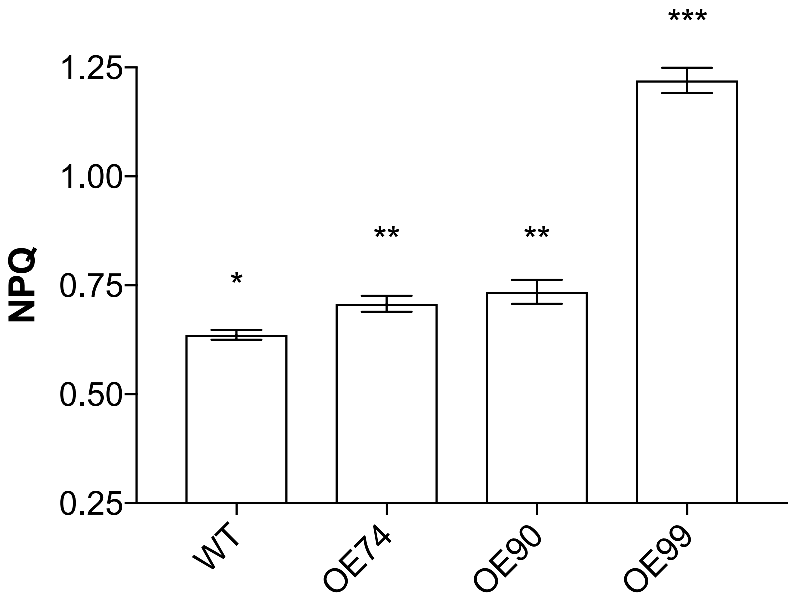

Supplementary Figure 9. Photoprotection in individual lines for experiment 3 in *psbS* over expressors and wild type rice. Data for individual lines from experiment 3 where non- photochemical quenching was measured over a 7-day period prior to anthesis at the top of the canopy using a monitoring fluorometer as described in materials and methods. Overexpressing (OE) and wild type (WT) are shown. The mean value for each day was recorded. Values are means from all plots and standard errors of the means (n=7). Asterisks indicate significant differences (P=0.0011, 0.6714, 0.8970 when comparing wild type and OE99, OE90 and OE74 respectively (one way analysis of variance)).

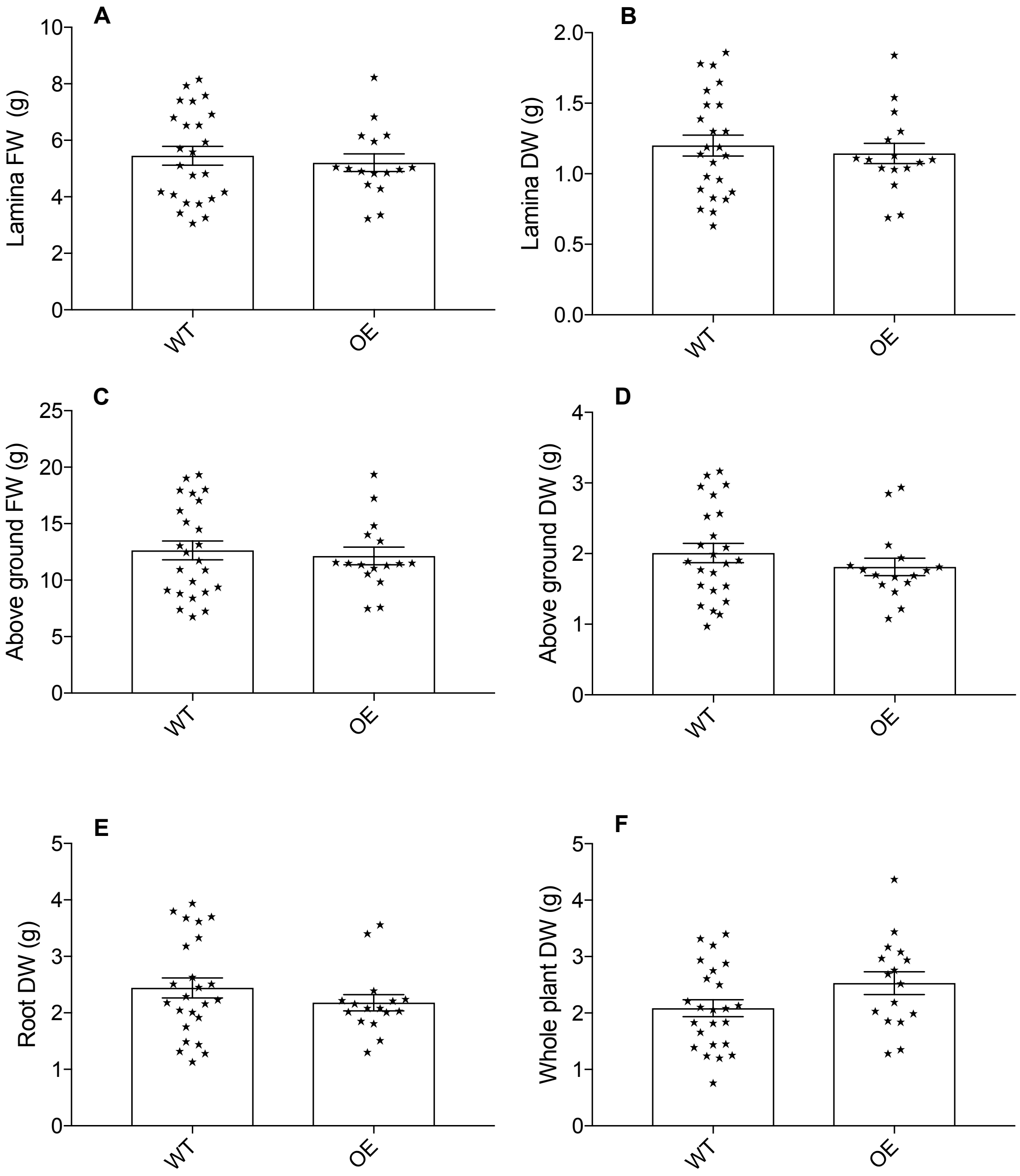

Supplementary Figure 10. Biomass and leaf area data in *psbS* over expressors and wild type rice when grown in artificial, static light. Harvest analysis of wild type (WT) and over expressing (OE) (3-16) lines grown under artificially static light (A) leaf lamina fresh weight (FW), (B) leaf Lamina DW, (C) Above ground FW, (D) Above ground dry weight (DW), (E) root DW, (F) Whole plant (root + shoot + stem (not shown) DW). Values are means of individual plants from all plots ± standard errors of the means for 2 independent experiments (n = 24). In all cases, differences between means were not significant except for whole plant DW (P=0.804, unpaired T test).

Supplementary references

[1] Hubbart, S., Ajigboye, O. O., Horton, P. & Murchie, E. H. The photoprotective protein PsbS exerts control over CO2 assimilation rate in fluctuating light in rice. *Plant J.* **71,** 402–412 (2012).
